# CX_3_CR1 differentiates F4/80^low^ monocytes into pro-inflammatory F4/80^high^ macrophages in the liver

**DOI:** 10.1038/s41598-018-33440-9

**Published:** 2018-10-10

**Authors:** Young-Sun Lee, Myung-Ho Kim, Hyon-Seung Yi, So Yeon Kim, Hee-Hoon Kim, Ji Hoon Kim, Jong Eun Yeon, Kwan Soo Byun, Jin-Seok Byun, Won-Il Jeong

**Affiliations:** 1 Department of Internal Medicine, Korea University College of Medicine, Seoul, 08308 Republic of Korea; 2 Lab of Liver Research, Graduate School of Medical Science and Engineering, KAIST, Daejeon, 34141 Republic of Korea; 3 Research Center for Endocrine and Metabolic Diseases, Chungnam National University School of Medicine, Daejeon, 34952 Republic of Korea; 4 Department of Oral Medicine, School of Dentistry, Kyungpook National University, Daegu, 41940 Republic of Korea

## Abstract

The expression of chemokine receptor CX_3_CR1 is related to migration and signaling in cells of the monocyte-macrophage lineage. The precise roles of CX_3_CR1 in the liver have been investigated but not clearly elucidated. Here, we investigated the roles of CX_3_CR1 in hepatic macrophages and liver injury. Hepatic and splenic CX_3_CR1^low^F4/80^low^ monocytes and CX_3_CR1^low^CD16^−^ monocytes were differentiated into CX_3_CR1^high^F4/80^high^ or CX_3_CR1^high^CD16^+^ macrophages by co-culture with endothelial cells. Moreover, CX_3_CL1 deficiency in human umbilical vein endothelial cells (HUVECs) attenuated the expression of interleukin-1β (IL-1β) and tumor necrosis factor-α (TNF-α), whereas recombinant CX_3_CL1 treatment reversed this expression in co-cultured monocytes. Upon treatment with clodronate liposome, hepatic F4/80^high^ macrophages were successfully depleted at day 2 and recovered similarly in CX_3_CR1^+/GFP^ and CX_3_CR1^GFP/GFP^ mice at week 4, suggesting a CX_3_CR1-independent replacement. However, F4/80^high^ macrophages of CX_3_CR1^+/GFP^ showed a stronger pro-inflammatory phenotype than CX_3_CR1^GFP/GFP^ mice. In clodronate-treated chimeric CX_3_CR1^+/GFP^ and CX_3_CR1^GFP/GFP^ mice, CX_3_CR1^+^F4/80^high^ macrophages showed higher expression of IL-1β and TNF-α than CX_3_CR1^−^F4/80^high^ macrophages. In alcoholic liver injury, despite the similar frequency of hepatic F4/80^high^ macrophages, CX_3_CR1^GFP/GFP^ mice showed reduced liver injury, hepatic fat accumulation, and inflammatory responses than CX_3_CR1^+/GFP^ mice. Thus, CX_3_CR1 could be a novel therapeutic target for pro-inflammatory macrophage-mediated liver injury.

## Introduction

The liver is considered an immunological organ because of the allocation of diverse types of innate and adaptive immune cells^1^. Kupffer cells are resident macrophages that are tightly attached along the liver sinusoidal endothelial cells (LSECs). Kupffer cells are characterized by the differential expression of specific markers (e.g. F4/80^high^CD11b^+^ or Ly6C^low^CD11b^+^ in mice but CD14^+^CD16^+^ in humans) compared to circulating F4/80^low^CD11b^+^ mouse and CD14^high^CD16^−^ human monocytes, and they participate in the pathogenesis of a broad spectrum of liver diseases and insulin resistance via switching of the alternative M2 phenotype to the classical M1 phenotype by various damage- and pathogen-associated molecular patterns^2,3^. The origin of Kupffer cells has been intensively studied but remains undefined. Fate-mapping recently revealed that several tissue-specific macrophages originate from either the yolk sac or fetal hematopoietic stem cells and proliferate locally^4–6^. Other findings indicate that Kupffer cells might originate from the bone marrow or intrahepatic precursor cells as a result of local proliferation^5,7–9^. However, the factors that regulate the differentiation of Kupffer cells from monocytes remain unknown.

Chemokines and their receptors play important roles in attracting circulating monocytes to inflamed liver tissues. CCR2-dependent migration of Ly6C^+^ monocytes in mice and CD14^+^ monocytes in humans constitute the predominant pro-inflammatory macrophages^10–12^. However, differential Ly6C expression in CCR2^+^ monocytes such as Ly6C^high^ monocytes (analogous to classical CD14^high^CD16^−^ human monocytes) and Ly6C^low^ monocytes (analogous to non-classical CD14^low^CD16^+^ human monocytes), play opposite (pro-inflammatory versus restorative) or dual roles in liver inflammation and fibrosis in mice and humans, or often simultaneously express M1 and M2 markers^12,13^. Circulating pro-inflammatory CCR2^high^CX_3_CR1^low^ monocytes differentiate into restorative CX_3_CR1^high^CCR2^low^ macrophages at sites of sterile liver injury. Ly6C^high^CX_3_CR1^low^ monocytes transform to CX_3_CR1^high^Ly6C^low^ macrophages that crawl along endothelial cells to dispose of their cellular debris. Both findings indicate protective functions of CX_3_CR1^high^ monocytes^11,14^. However, the mechanisms by which CX_3_CR1 expression is up-regulated in these conditions and its role have not been investigated.

CX_3_CR1 is a chemokine receptor of fractalkine (CX_3_CL1) that is expressed on various cells of the macrophage lineage^5,15^. CX_3_CR1 is also involved in the colonization of pre-macrophages at the embryonic stage, leading to differentiation of tissue-resident macrophages, including Kupffer cells^15^. However, CX_3_CR1 expression is absent in adult Kupffer cells^5^. Functionally, CX_3_CL1-CX_3_CR1 interaction is important for the migration of immune cells, blood monocyte homeostasis, and for cellular signaling processes that include stimulation of insulin secretion from β-cells^16–18^. Additionally, CX_3_CR1 deficiency aggravates liver inflammation and fibrosis by up-regulating the expressions of tumor necrosis factor-α (TNF-α) and transforming growth factor-β (TGF-β), and increasing the infiltration of inflammatory monocytes^19,20^. However, the role of CX_3_CR1 in alcoholic liver disease has not been studied.

Interestingly, CX_3_CL1 is synthesized from various types of liver cells, which include hepatocytes, hepatic stellate cells (HSCs), and LSECs in chronic liver diseases of humans and mice^20–22^. In endothelial cells, CX_3_CL1 expression is up-regulated mainly by cytokine stimulation and is reportedly induced by changes in shear stress^23^. CX_3_CR1-expressing monocytes do not dislodge once bound to CX_3_CL1^24^, supporting the settlement theory of Kupffer cells. However, whether CX_3_CR1 is involved in differentiation of circulating monocytes into resident-like macrophages in the liver remains unclear.

In the present study, we investigated the role of CX_3_CR1 in the functional and phenotypic polarization of hepatic macrophages from circulating monocytes using an *in vitro* differentiation model and an *in vivo* alcoholic liver disease model.

## Results

### Hepatic endothelial cells regulate the expression of F4/80 and CX_3_CR1 in hepatic monocytes

**Figure 1:**
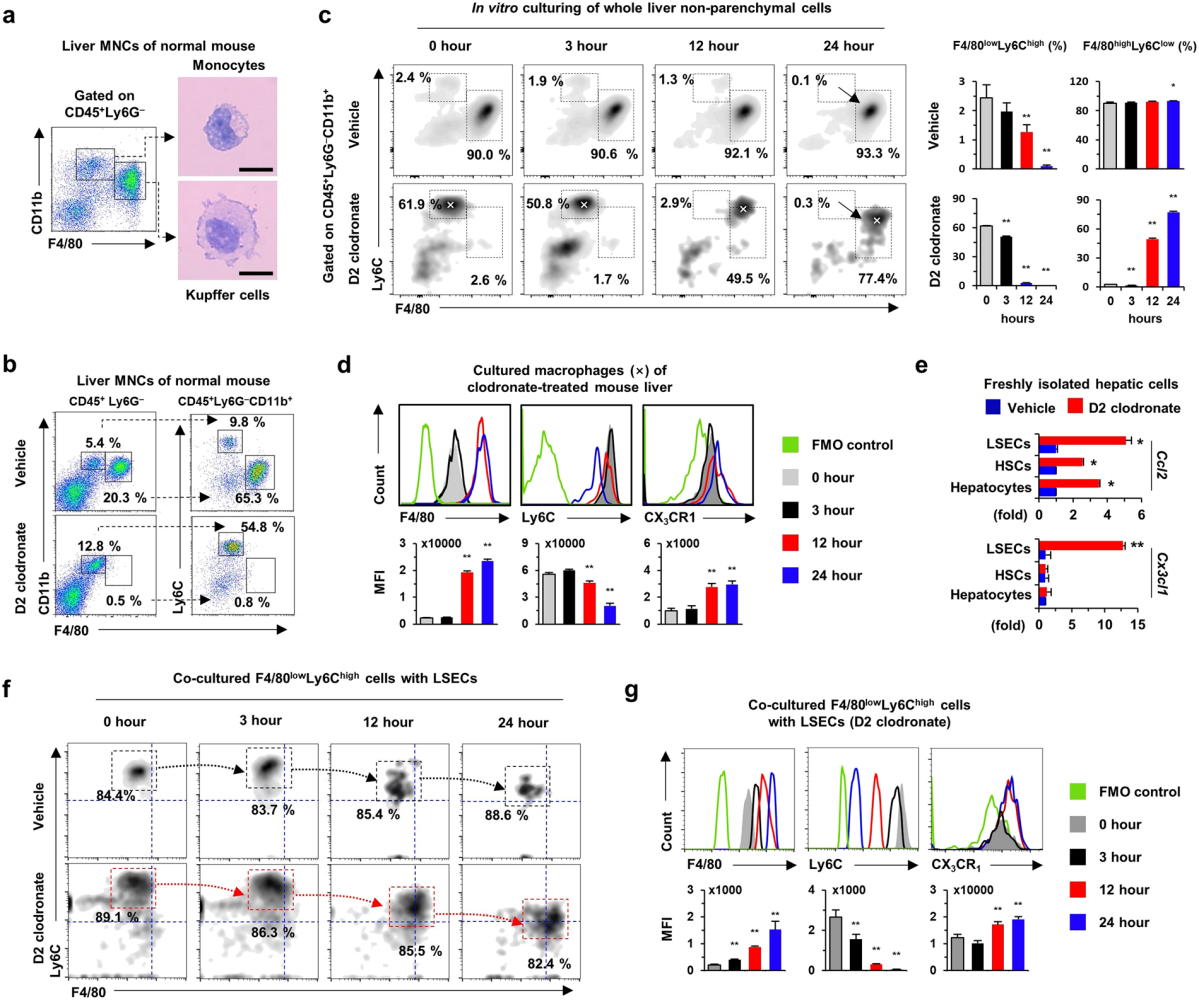
Liver sinusoidal endothelial cells (LSECs) differentiate F4/80^low^Ly6C^high^CX_3_CR1^low^ monocytes into F4/80^high^Ly6C^low^CX_3_CR1^high^ Kupffer-like macrophages. (**a**) Isolated mononuclear cells (MNCs) were subjected to flow cytometry. Monocytes and Kupffer cells were subjected to Giemsa staining. Bar = 10 μm. (**b**) Kupffer cells were depleted by clodronate liposome treatment for 2 days (D2 clodronate). (**c**,**d**) Untreated whole liver non-parenchymal cells or cells treated with D2 clodronate were cultured *in vitro* for 24 h and analyzed by flow cytometry. (**e**) Gene expression was analyzed in isolated LSECs, hepatic stellate cells, and hepatocytes. Data are expressed as the mean ± SEM. *p < 0.05, **p < 0.01 compared to corresponding control. (**f**,**g**) Isolated F4/80^low^Ly6C^high^ monocytes were co-cultured with LSECs of vehicle or D2 clodronate-treated mice for 24 h and they were subjected to flow cytometry. Data are expressed as the mean ± SEM. *p < 0.05, **p < 0.01 compared to 0 h. The results represent three independent experiments.

Circulating F4/80^low^CD11b^+^ monocytes had bean-shaped nuclei and scant cytoplasm, whereas resident F4/80^high^CD11b^+^ Kupffer cells displayed abundant cytoplasm with deviated nuclei in healthy mouse livers (Fig. 1a and Supplementary Fig. 1). After Kupffer cell depletion by clodronate treatment, the circulating monocytes were further characterized by high levels of Ly6C (F4/80^low^CD11b^+^Ly6C^high^), unlike those in F4/80^high^CD11b^+^Ly6C^low^ Kupffer cells (Fig. 1b). To identify the characteristics of these cells *in vitro*, non-parenchymal cells were isolated from livers of mice with and without 2-day clodronate liposome treatment (D2 clodronate) and the cells were cultured *in vitro* for 24 h. Similar to *in vivo* results, unique populations of monocytes and Kupffer cells were identified at 0 h, whereas frequencies of F4/80^low^Ly6C^high^ monocytes in vehicle-treated mice gradually decreased and were absent at 24 h (Fig. 1c). More interestingly, *in vitro* culturing transformed the phenotype of F4/80^low^Ly6C^high^ monocytes into F4/80^high^Ly6C^low^ Kupffer-like cells in D2 clodronate-treated mice (Fig. 1c). A histogram analysis showed that Ly6C expression decreased, but the expression of F4/80 and CX_3_CR1 gradually increased in F4/80^low^Ly6C^high^ monocytes (Fig. 1d). Quantitative reverse transcription PCR (qRT-PCR) analyses showed that clodronate treatment significantly increased the expression of CX_3_CL1 only in LSECs, in contrast to increased expression of CCL2 in hepatocytes, HSCs, and LSECs (Fig. 1e), suggesting possible interaction between F4/80^low^Ly6C^high^ monocytes and CX_3_CL1^+^ LSECs. To confirm this suggestion, F4/80^low^Ly6C^high^ monocytes were co-incubated with LSECs obtained from D2 clodronate-treated mouse liver. After co-culturing, F4/80^low^Ly6C^high^ monocytes (85.7 ± 2.3%) had markedly differentiated into F4/80^high^Ly6C^low^ macrophages by LSECs from clodronate-treated mice compared to LSECs from vehicle-treated mice (Fig. 1f). Along with the altered expression of F4/80 and Ly6C, CX_3_CR1 expression was significantly increased by co-culture with CX_3_CL1^+^ LSECs (Fig. 1g). These findings suggested that LSECs with CX_3_CL1 expression could promote the differentiation of F4/80^low^Ly6C^high^ monocytes into CX_3_CR1^high^F4/80^high^Ly6C^low^ macrophages.

### LSECs regulate F4/80 and CX_3_CR1 expression in splenic monocytes

**Figure 2:**
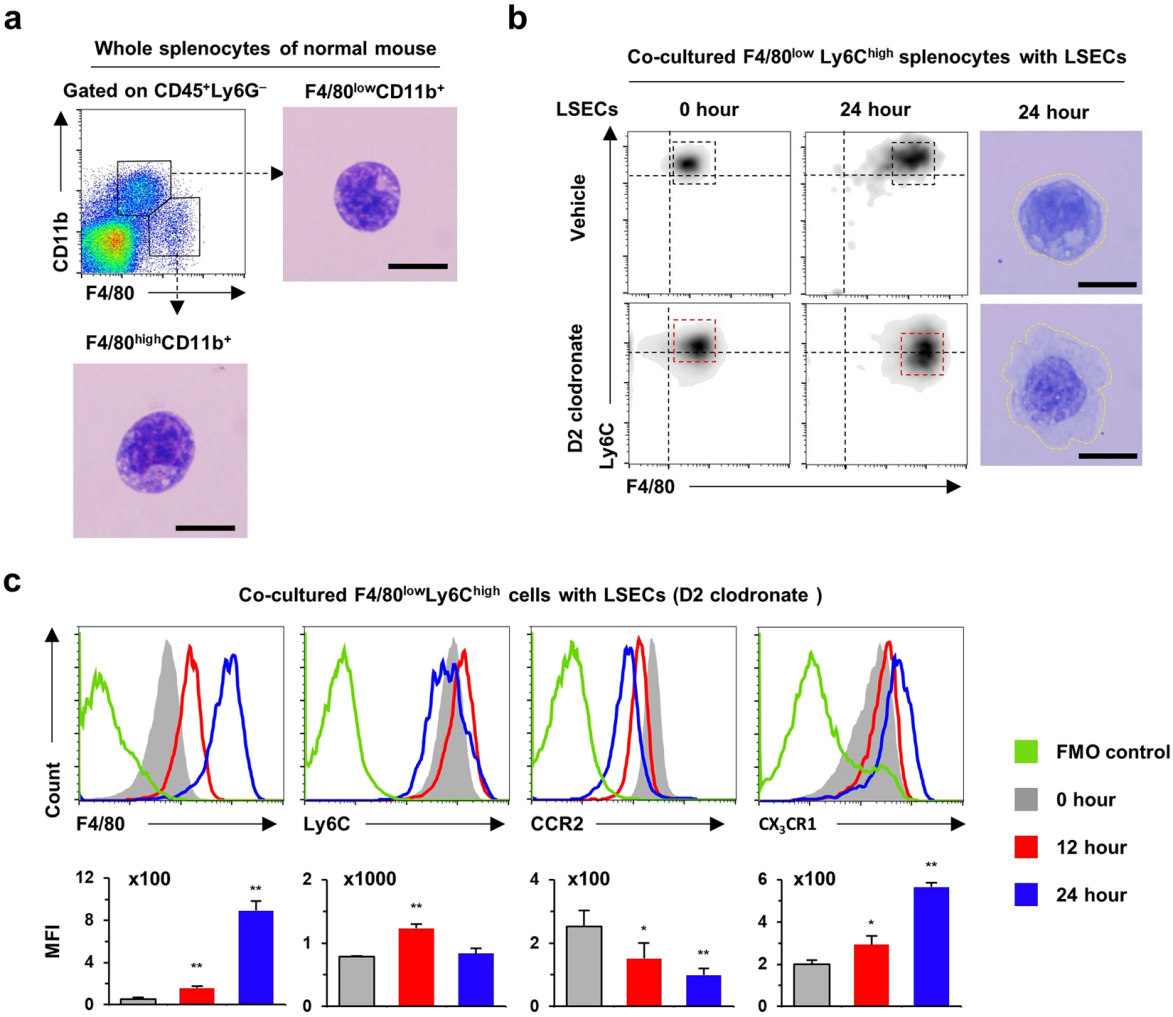
Liver sinusoidal endothelial cells up-regulate expression of F4/80 and CX_3_CR1 in splenic Ly6C^+^ monocytes. (**a**) Isolated F4/80^low^CD11b^+^ monocytes and F4/80^high^CD11b^+^ macrophages of spleen were subjected to Giemsa staining. Bar = 10 μm. (**b**,**c**) Splenic F4/80^low^Ly6C^+^ monocytes were co-cultured with LSECs of vehicle or D2 clodronate-treated mice for 24 h. They were subjected to flow cytometry and Giemsa staining. Data are expressed as the mean ± SEM. *p < 0.05, **p < 0.01 compared to 0 h. The results represent three independent experiments.

To confirm whether differentiation of splenic F4/80^low^ monocytes into F4/80^high^ macrophages could be reproducible similar to circulating blood F4/80^low^ monocytes in the liver, splenic F4/80^low^ monocytes were co-cultured with LSECs of D2 clodronate-treated mouse liver. Flow cytometry analysis revealed that freshly isolated splenic F4/80^low^CD11b^+^ monocytes had morphology similar to that of hepatic F4/80^low^CD11b^+^ monocytes, while splenic resident F4/80^high^CD11b^+^ macrophages had a smaller cytoplasm than F4/80^high^CD11b^+^ Kupffer cells (Fig. 2a). However, intriguingly, co-culturing with LSECs gradually increased the expression of F4/80 in splenic F4/80^low^CD11b^+^ monocytes and their morphology resembled the appearance of F4/80^high^CD11b^+^ Kupffer cells (Fig. 2b). Histogram analysis showed that co-culturing with LSECs increased the expression of F4/80 and CX_3_CR1, whereas the expression of CCR2 and Ly6C decreased in co-cultured splenic F4/80^high^CD11b^+^ monocytes at 24 h (Fig. 2c). These findings suggested that LSECs are important in the differentiation of CX_3_CR1^low^F4/80^low^ monocytes into CX_3_CR1^high^F4/80^high^ macrophages.

### HUVECs regulate phenotypes of human classical CD14^high^CD16^−^ monocytes

**Figure 3:**
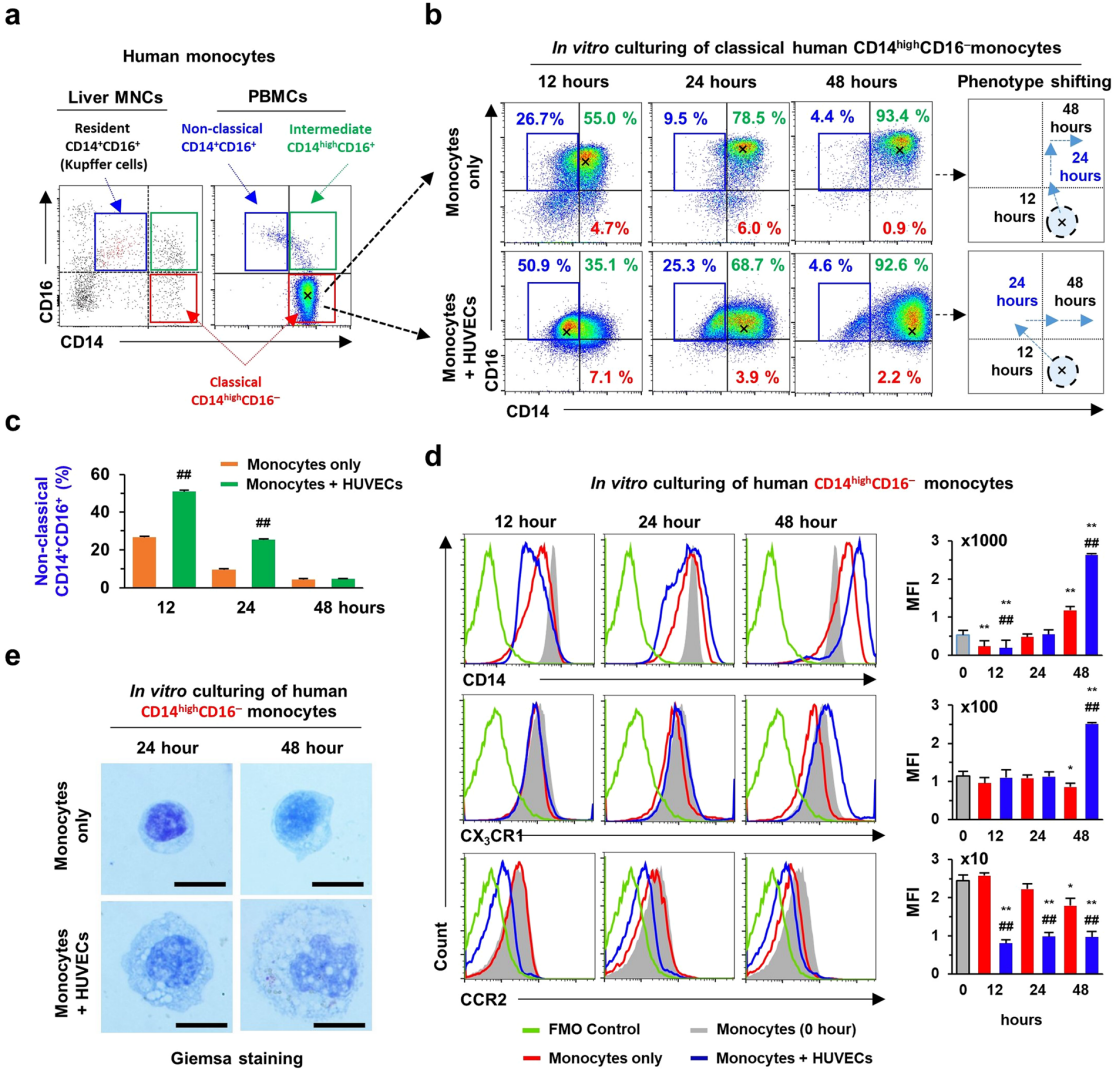
Human umbilical vein endothelial cells (HUVECs) differentiate human CD14^high^CD16^−^ monocytes into CD14^+^CD16^+^ monocytes along with high expression of CX_3_CR1. (**a**) Specific monocyte subsets were separated in human liver MNCs and peripheral blood mononuclear cells (PBMCs). (**b**,**c**) Human CD14^high^CD16^−^ monocytes were co-cultured with HUVECs for 48 h. Then, their phenotypic shifts with respect to the expression of CD16 and CD14 were analyzed by flow cytometry and indicated by arrows and asterisks. Data are expressed as the mean ± SEM. ^#^*p* < 0.05, ^##^*p* < 0.01 compared to corresponding monocytes only (**d**) Expression of CD14, CX_3_CR1 and CCR2 in cultured CD14^high^CD16^−^ monocytes were analyzed by flow cytometry and compared to those of human liver monocyte subsets. Data are expressed as the mean ± SEM. *p < 0.05, **p < 0.01 compared to 0 h. ^#^*p* < 0.05, ^##^*p* < 0.01 compared to corresponding monocytes only. (**e**) Morphological changes of co-cultured CD14^high^CD16^−^ monocytes were assessed after Giemsa staining. Bar = 10 μm. The results represent three independent experiments.

Next, we determined whether CX_3_CL1^+^ endothelial cells drive the differentiation of human CD14^high^CD16^−^ monocytes into CX_3_CR1^high^CD14^high^CD16^+^ macrophages. As previously reported^18^, flow cytometry revealed three subsets of human monocytes in human peripheral blood mononuclear cells (PBMCs) and liver mononuclear cells (MNCs): classical, intermediate, and non-classical monocytes, were detected (Fig. 3a and Supplementary Fig. 2a). Therefore, isolated CD14^high^CD16^−^ monocytes were co-cultured with HUVECs for 48 h. During *in vitro* co-culturing, most classical CD14^high^CD16^−^ monocytes shifted towards intermediate CD14^high^CD16^+^ monocytes with or without co-culturing of HUVECs at 48 h (Fig. 3b and Supplementary Fig. 2b). Unlike culturing of only monocytes, classical CD14^high^CD16^−^ monocytes temporarily shifted to CD14^+^CD16^+^ non-classical phenotype after being co-cultured with HUVECs at 12 h and further shifted towards the intermediate phenotype at 24 h (Fig. 3b,c). Histogram analysis revealed that CX_3_CR1 expression increased significantly in CD14^high^CD16^−^ monocytes co-cultured with HUVECs but decreased in classical monocytes cultured without HUVECs (Fig. 3d and Supplementary Fig. 2c). However, CCR2 expression was reduced in both monocytes cultured alone or with HUVECs, whereas expression of CD68 and CD206 was further increased in monocytes cultured in the absence of HUVECs than in the presence of HUVECs (Fig. 3d and Supplementary Fig. 2d). Morphological observations showed that freshly isolated human blood monocytes had large and non-lobulated nuclei with scant cytoplasm (Supplementary Fig. 2a), whereas the cytoplasm volume in monocytes increased gradually during *in vitro* culture, and nuclei of cells became more peripherally located (Fig. 3e). These observations were more significant in monocytes co-cultured with HUVECs (Fig. 3e). These observations suggested that classical CD14^high^CD16^−^ monocytes differentiate towards the intermediate or non-classical CX_3_CR1^+^CD14^+^CD16^+^ macrophages during co-culturing with HUVECs.

### CX_3_CL1 of HUVECs regulates inflammatory phenotypes of human CD14^high^CD16^−^ monocytes

**Figure 4:**
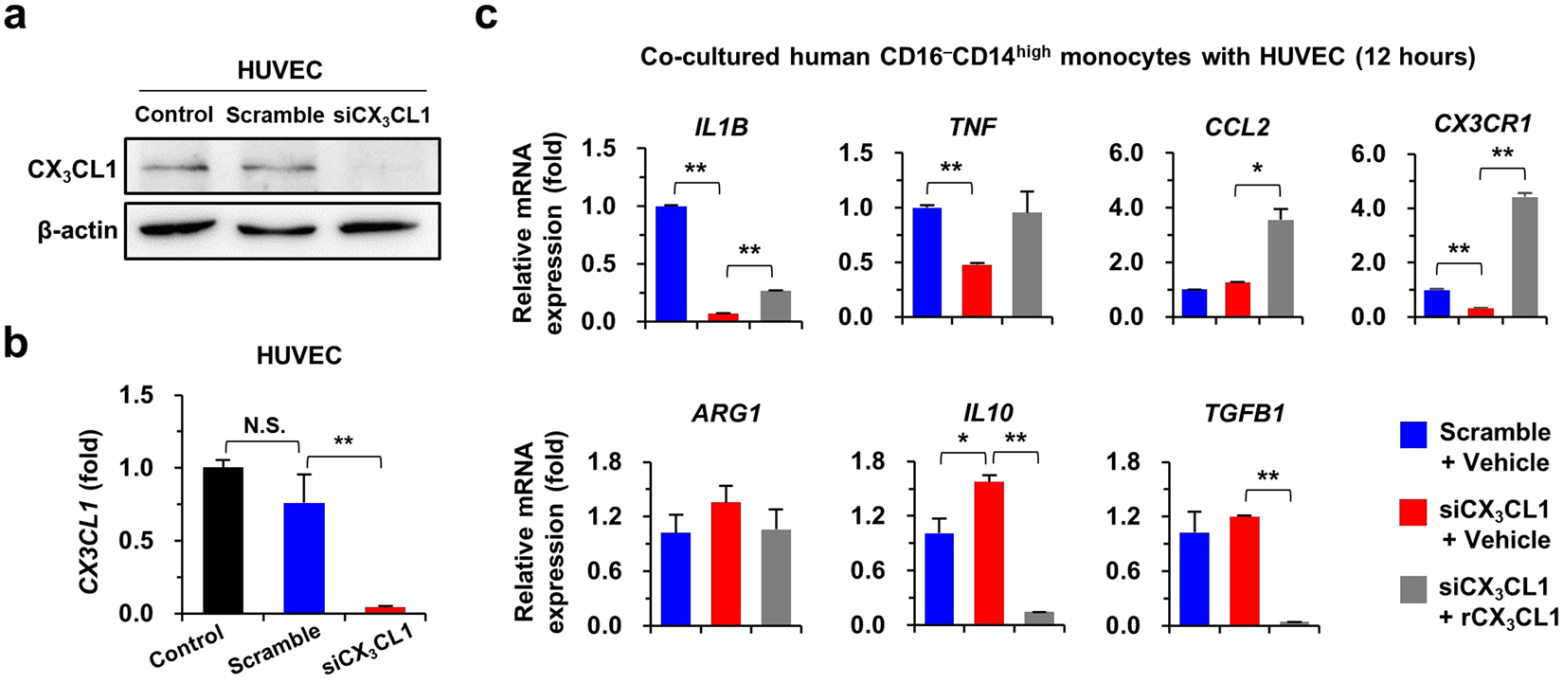
CX_3_CL1 of HUVECs promote pro-inflammatory phenotype of human CD14^high^CD16^−^ monocytes. (**a**,**b**) Silencing of CX_**3**_CL1 expression was assessed by western blot (**a**) and quantitative reverse transcription PCR (qRT-PCR) (**b**) in HUVECs after small interfering RNA (siRNA) transfection. Original western blots are shown in Supplementary Fig. 3. (**c**) CD14^high^CD16^−^ monocytes of human PBMCs were co-cultured with HUVECs for 12 h with or without treatment of recombinant CX_**3**_CL1. Co-cultured CD14^high^CD16^−^ monocytes were subjected to qRT-PCR analysis. Data are expressed as the mean ± SEM. *p < 0.05, **p < 0.01 compared to corresponding control. The results represent three independent experiments.

Based on the preceding findings, we investigated whether HUVEC-derived CX_3_CL1 regulates the inflammatory phenotype of human CD14^high^CD16^−^ monocytes. siRNA was used to silence CX_3_CL1 expression in HUVECs (Fig. 4a,b). CD14^high^CD16^−^ monocytes co-cultured with CX_3_CL1-depleted HUVECs showed decreased expression of pro-inflammatory genes (*IL1B* and *TNF*), but slight increases in the expression of anti-inflammatory genes (*ARG1, IL10*, and *TGFB1*) (Fig. 4c). In contrast, treatment with recombinant CX_3_CL1 reversed the expression of pro- and anti-inflammatory genes in co-cultured monocytes (Fig. 4c). These results suggested that CX_3_CL1 of HUVECs regulates inflammatory signals of co-cultured CD14^high^CD16^−^ monocytes.

### CX_3_CR1 expression regulates inflammatory phenotypes of F4/80^high^CD11b^+^ macrophages in the liver

**Figure 5:**
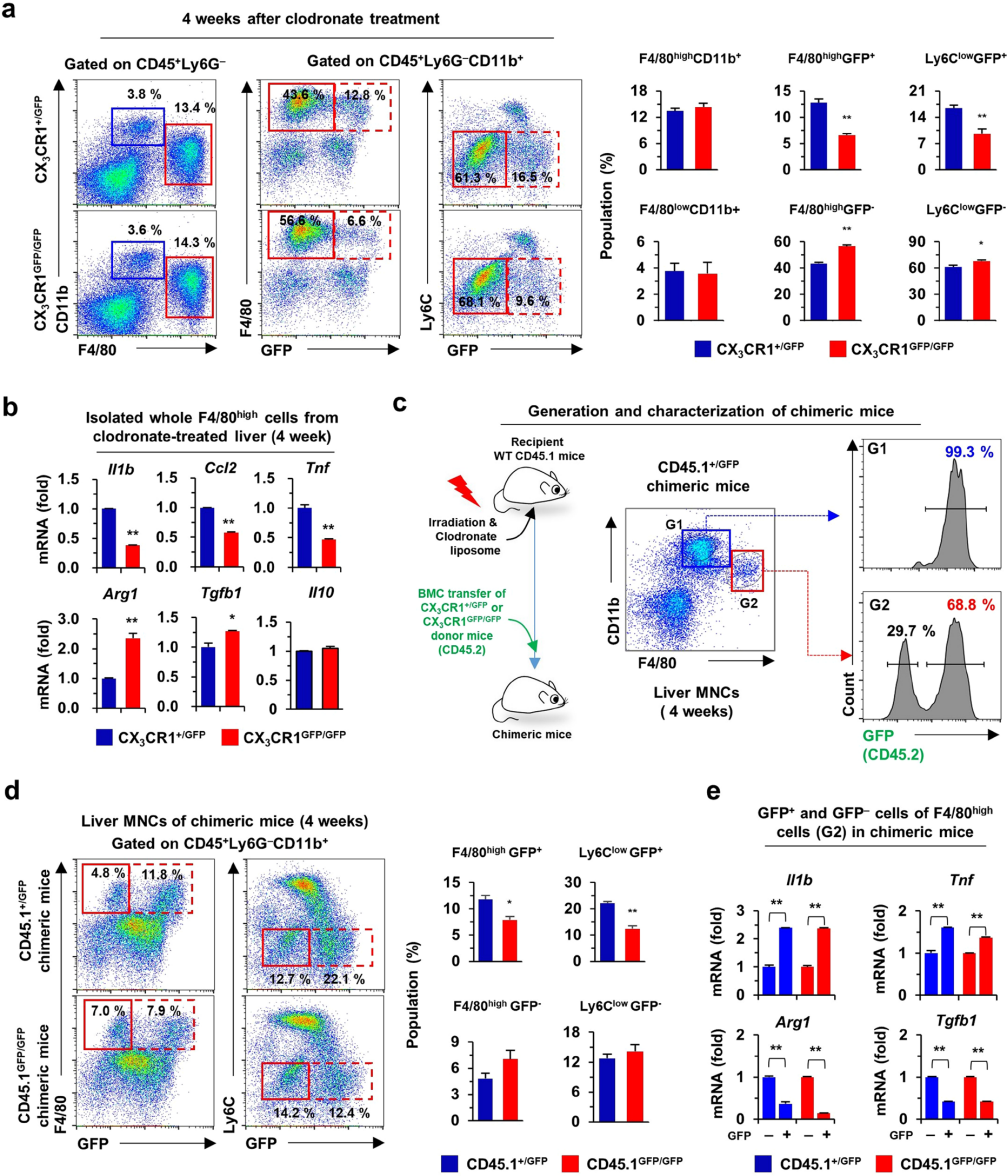
CX_3_CR1 expression assigns two subsets and pro-inflammatory phenotype to F4/80^high^ or Ly6C^low^ macrophages in the liver. (**a**) Liver monocytes and macrophages were analyzed by flow cytometry using isolated liver MNCs of CX_3_CR1^+/GFP^ and CX_3_CR1^GFP/GFP^ mice and 4-week after clodronate liposome treatment. (**b**) Isolated whole F4/80^high^ cells were subjected to qRT-PCR analysis at week 4 after clodronate liposome treatment. (**c**) To generate chimeric mice, recipient wild type mice (CD45.1) were transplanted with donor bone marrow cells (BMC) of CX_3_CR1^+/GFP^ or CX_3_CR1^GFP/GFP^ mice (CD45.2) after irradiation and clodronate liposome treatment. Chimerism was assessed by expression of GFP (CD45.2) in liver MNCs of recipient mice. (**d**) Isolated liver MNCs from chimeric mice were subjected to flow cytometry. (**e**) F4/80^high^GFP^+^ and F4/80^high^GFP^−^ cells of chimeric mice were subjected to qRT-PCR analysis. Data are expressed as the mean ± SEM. *p < 0.05, **p < 0.01 compared to GFP^−^ F4/80^high^ cells. The results represent two independent experiments.

Next, we investigated the differentiation and pro-inflammatory roles of CX_3_CR1 in F4/80^+^CD11b^+^ macrophage subsets (F4/80^high^ and F4/80^low^) of liver using clodronate liposome-treated CX_3_CR1 knock-in mice (CX_3_CR1^+/*GFP*^ and CX_3_CR1^*GFP/GFP*^). In normal liver, similar frequencies of GFP^+^ (*Cx3cr1* gene-expressing) cells were detected in F4/80^high^ and F4/80^low^ macrophage subsets of both CX_3_CR1^+/*GFP*^ and CX_3_CR1^*GFP/GFP*^ mice. By contrast the number of GFP^+^ cells were significantly increased in a F4/80^low^ macrophage subset in both CX_3_CR1^+/*GFP*^ and CX_3_CR1^*GFP/GFP*^ mice after clodronate liposome-induced depletion of resident F4/80^+^ macrophages (Supplementary Fig. 4a,b). At 4 weeks, the depleted F4/80^high^ macrophage subset recovered to a similar extent in both CX_3_CR1 knock-in mice, suggesting that CX_3_CR1 is not critical for replacement of the resident F4/80^high^ macrophage subset (Fig. 5a and Supplementary Fig. 4c). However, the frequencies of GFP^+^ cells in a F4/80^high^ macrophage subset (or Ly6C^low^) were significantly reduced in CX_3_CR1^*GFP/GFP*^ mice compared to those in CX_3_CR1^+/*GFP*^ mice (Fig. 5a). qRT-PCR analysis demonstrated the reduced expression of *Il1b, Ccl2*, and *Tnf*, whereas the expression of *Arg1 and Tgfb1* was significantly elevated in a F4/80^high^ macrophage subset of CX_3_CR1^*GFP/GFP*^ mice compared to those of CX_3_CR1^+/*GFP*^ mice (Fig. 5b). The findings suggested that high frequencies of CX_3_CR1-expressing cells might affect the elevated expression of pro-inflammatory genes in a F4/80^high^ macrophage subset during replacement of F4/80^+^ macrophage subsets. To further confirm this suggestion, chimeric mice (recipient WT mice with CD45.1 expression) were generated by transplantation of bone marrow cells of CX_3_CR1^+/*GFP*^ and CX_3_CR1^*GFP/GFP*^ mice (with expression of CD45.2) after irradiation plus clodronate liposome treatment (Fig. 5c). At week 4, chimeric mice were euthanized to assess the chimerism of hepatic F4/80^+^ macrophages. Flow cytometry revealed that almost all (99.3 ± 0.6%) F4/80^low^CD11b^+^ monocytes (G1 gating) were replaced from the GFP^+^ donor bone marrow cells, whereas GFP^+^CD45.2^+^ (68.8 ± 0.8%) and GFP^−^CD45.1^+^ (29.7 ± 0.5%) F4/80^high^ CD11b^+^ macrophages (G2 gating) were replaced from donor and recipient mice, respectively (Fig. 5c). More interestingly, the number of GFP^+^ cells of a F4/80^high^ macrophage subset (Ly6C^low^ macrophages) were significantly increased in CD45.1^+/*GFP*^ chimeric mice compared to CD45.1^*GFP/GFP*^ chimeric mice (Fig. 5d). Moreover, GFP^+^F4/80^high^ macrophages showed an increased pro-inflammatory phenotype compared to GFP^−^F4/80^high^ macrophages in both CD45.1^+/*GFP*^ and CD45.1^*GFP/GFP*^ chimeric mice (Fig. 5e), suggesting that the frequencies of CX_3_CR1-expressing cells among the F4/80^high^ macrophage subset might influence inflammation in liver injury.

### CX_3_CR1 deficiency attenuates ethanol-induced liver injury

**Figure 6:**
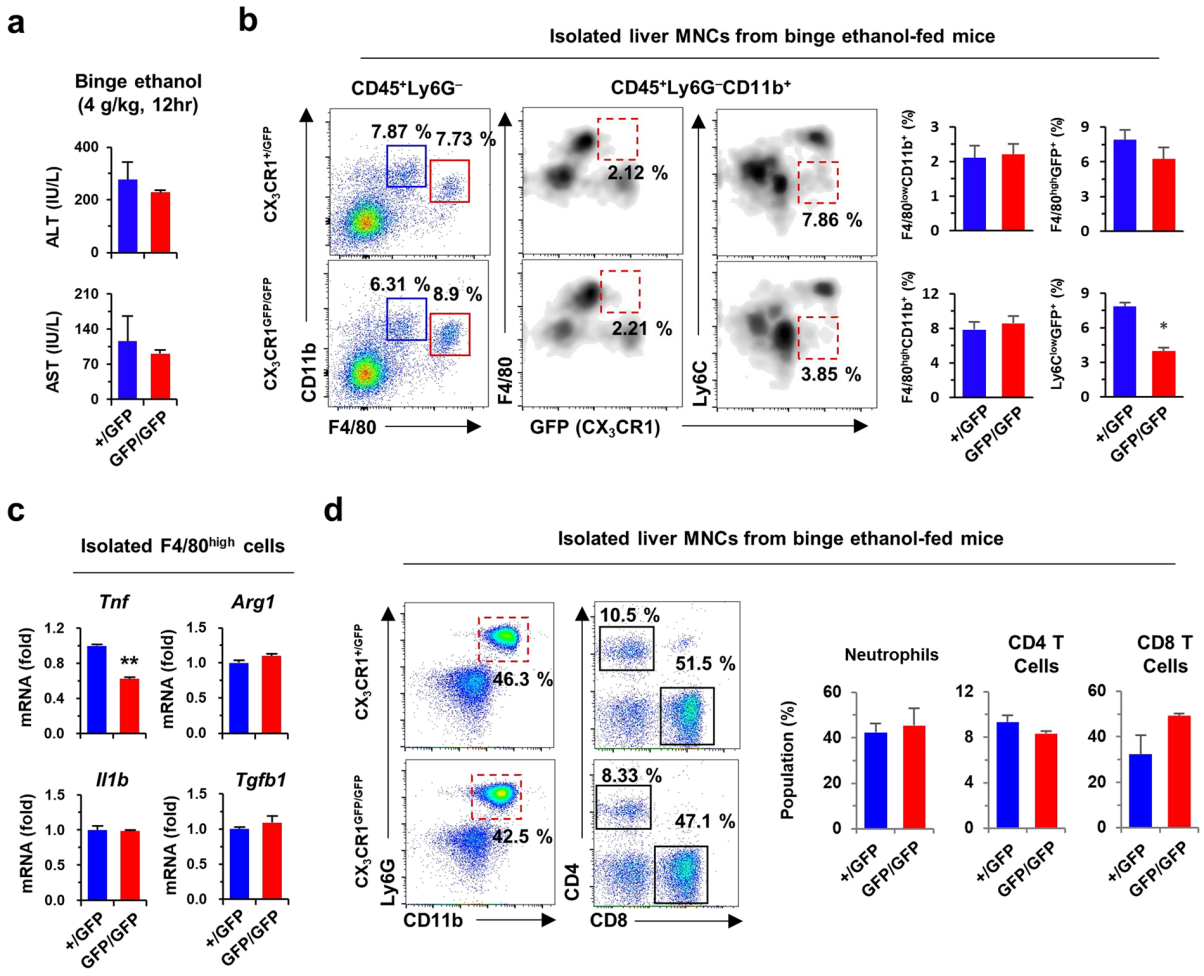
CX_3_CR1 deficiency attenuates acute alcoholic liver injury. To induce acute alcoholic liver injury, CX_3_CR1^+/GFP^ (n = 4) and CX_3_CR1^GFP/GFP^ (n = 4) mice were fed 4 g/kg ethanol (binge ethanol drinking). (**a**) Serum levels of ALT and AST were measured in binge ethanol-fed mice. (**b**,**c**) Isolated liver MNCs and F4/80^high^ cells from binge ethanol-fed mice were subjected to flow cytometry and qRT-PCR analysis, respectively. Data are expressed as the mean ± SEM. *p < 0.05, **p < 0.01 compared to corresponding controls. (**d**) Isolated liver MNCs from binge ethanol-fed mice were subjected to flow cytometry. The results represent two independent experiments.

**Figure 7:**
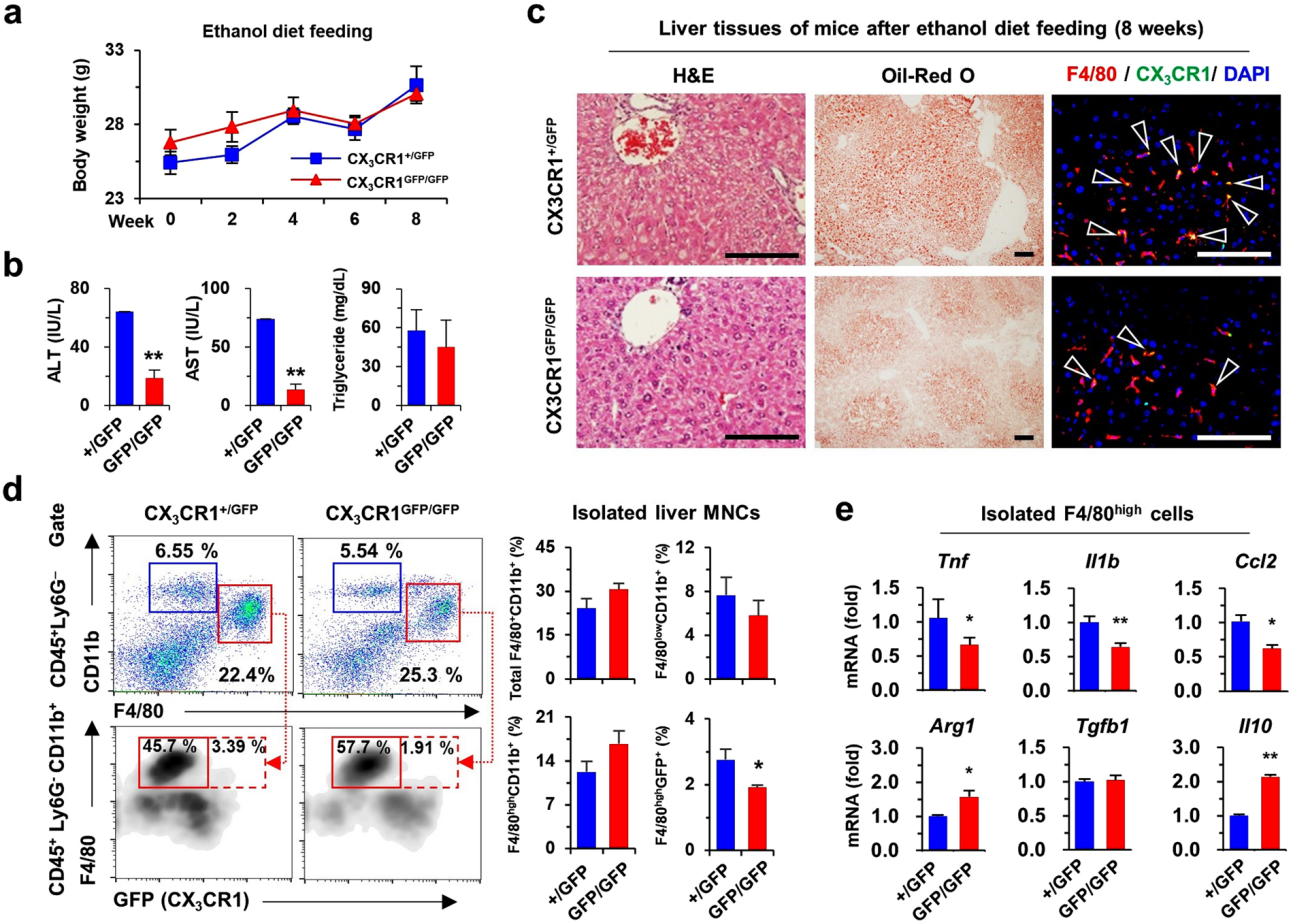
CX_3_CR1 deficiency attenuates alcoholic liver injury. CX_3_CR1^+/GFP^ (n = 4) and CX_3_CR1^GFP/GFP^ (n = 4) mice were fed 5% liquid ethanol diet for 8 weeks. (**a**,**b**) Body weight changes and serum levels of ALT, AST, and triglyceride were measured. (**c**) Liver sections were stained with hematoxylin and eosin (H & E) and Oil-Red O, or with F4/80 antibody. Bar = 100 μm. (**d**) Isolated liver MNCs were subjected to flow cytometry. (**e**) Isolated F4/80^high^ cells were subjected to qRT-PCR analysis. Data are expressed as the mean ± SEM. *p < 0.05, **p < 0.01 compared to corresponding controls. The results represent two independent experiments.

To investigate the pro-inflammatory effect of CX_3_CR1^+^F4/80^high^ macrophages, liver injury was induced by binge ethanol drinking or an 8-week diet of liquid ethanol in CX_3_CR1^+/*GFP*^ and CX_3_CR1^*GFP/GFP*^ mice. Acute liver injury was not associated with significant differences in serum levels of alanine aminotransferase (ALT) and aspartate aminotransferase (AST), and altered frequencies of F4/80^high^CD11b^+^ Kupffer cells including CX_3_CR1^+^F4/80^high^ cells between CX_3_CR1^+/*GFP*^ and CX_3_CR1^*GFP/GFP*^ mice (Fig. 6a,b and Supplementary Fig. 5a). However, CX_3_CR1^*GFP/GFP*^ mice showed lower expression of TNF-α than CX_3_CR1^+/*GFP*^ mice, although similar hepatic expression of CX_3_CL1 in response to acute liver injury was evident (Fig. 6c and Supplementary Fig. 5b). Additionally, there were no differences in the frequencies of Ly6G^+^CD11b^+^ neutrophils, CD4^+^ T cells, and CD8^+^ T cells in CX_3_CR1^+/*GFP*^ and CX_3_CR1^*GFP/GFP*^ mice (Fig. 6d). The chronic ethanol diet did not result in significant changes in the body weights of CX_3_CR1^*GFP/GFP*^ and CX_3_CR1^+/*GFP*^ mice (Fig. 7a), but serum levels of ALT and AST in CX_3_CR1-deficient (CX_3_CR1^*GFP/GFP*^) mice was significantly reduced compared to CX_3_CR1^+/*GFP*^ mice (Fig. 7b). Histological observations revealed reduced fat accumulation and infiltration of CX_3_CR1^+^F4/80^+^ cells in the livers of CX_3_CR1^*GFP/GFP*^ mice compared to CX_3_CR1^+/*GFP*^ mice (Fig. 7c and Supplementary Fig. 5c). Flow cytometry analyses revealed similar frequencies of total F4/80^+^CD11b^+^ cells in CX_3_CR1^*GFP/GFP*^ and CX_3_CR1^+/*GFP*^ mice (Fig. 7d). However, pro-inflammatory CX_3_CR1^+^ cells of F4/80^high^ macrophages (CX_3_CR1^+^F4/80^high^) were significantly lower in the livers of CX_3_CR1^*GFP/GFP*^ mice than in CX_3_CR1^+/*GFP*^ mice, although there was no difference in the frequency of F4/80^high^CD11b^+^ macrophages between both mice (Fig. 7d). F4/80^high^ macrophages of CX_3_CR1^+/*GFP*^ mice displayed a more pro-inflammatory phenotype than CX_3_CR1^*GFP/GFP*^ mice (Fig. 7e). However, frequencies of other immune cells including CD4^+^ T cells, CD8^+^ T Cells, NK cells, dendritic cells and neutrophils were similar, and expression of *Ifng*, *Il17*, *Il4*, and *Il13* in liver MNCs was not different between both mice (Supplementary Fig. 5d,e). Consistent with these observations, the increased expression of *Cx3cl1* and *Cx3cr1* in the GEO database (GSE97234) was significantly associated with high expression of *Il1β* and *Tnf* in mice with alcoholic steatohepatitis compared to healthy control mice (Supplementary Fig. 6a,b). These observations suggested that pro-inflammatory CX_3_CR1^+^F4/80^high^ macrophages contribute to alcoholic liver injury.

## Discussion

**Figure 8:**
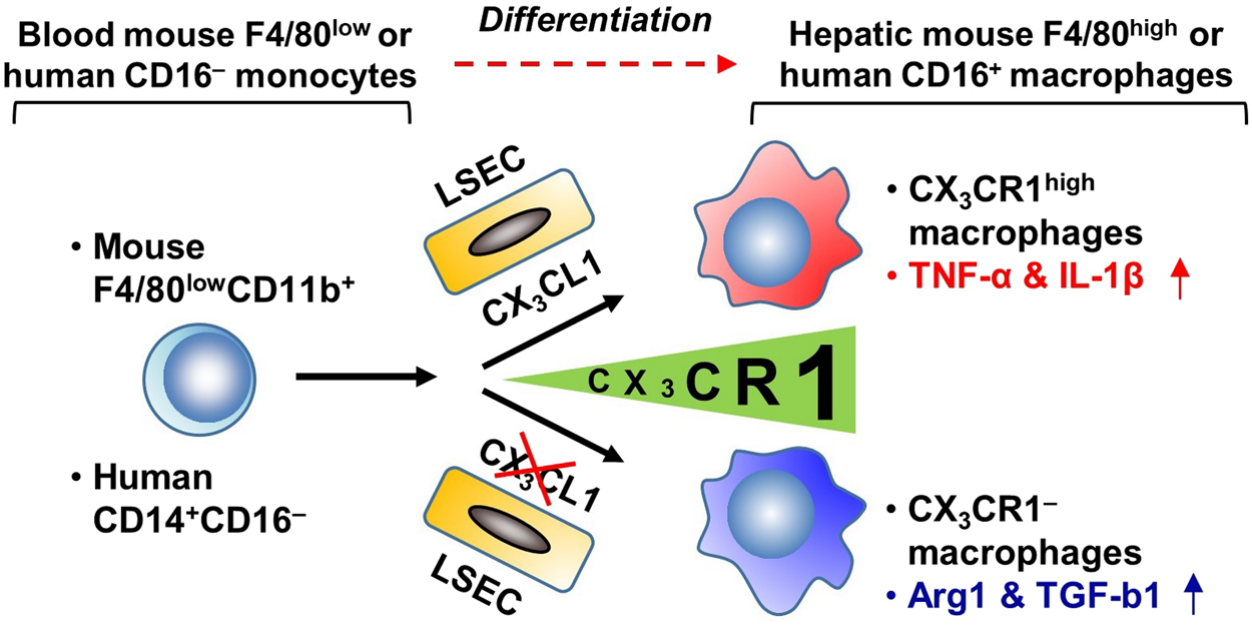
Schematic summary. CX_3_CR1 differentiates mouse monocytes (F4/80^low^CD11b^+^) and human monocytes (CD14^+^CD16^−^) into pro-inflammatory macrophages. Liver sinusoidal endothelial cells (LSECs) express CX_3_CL1 in the liver.

Recent developments in gene editing techniques have enabled fate-map tracing of tissue-resident macrophages. Nevertheless, most studies have focused on the origins of macrophages^4–6^. In the present study, we investigated whether CX_3_CR1 might be involved in the differentiation of circulating blood monocytes into hepatic resident macrophages in mice and humans. We observed that F4/80^low^CX_3_CR1^low^ monocytes were converted into pro-inflammatory F4/80^high^CX_3_CR1^high^ macrophages via interaction with endothelial cells *in vitro*, and that CX_3_CR1 deficiency ameliorated alcoholic liver injury in mice by decreasing the number of pro-inflammatory F4/80^high^CX_3_CR1^high^ macrophages (Fig. 8).

There are at least two distinct monocyte subsets in humans and mice: the classical mouse Ly6C^high^ or human CD14^high^CD16^−^ monocytes and non-classical mouse Ly6C^low^ or human CD14^low^CD16^+^ monocytes^25^. The non-classical monocytes express higher levels of CX_3_CR1 and lower levels of CCR2 than classical monocytes in both mice and humans^18,25^. These non-classical Ly6C^low^ and CD14^low^CD16^+^ monocytes contribute to the maintenance of endothelial integrity, whereas classical Ly6C^+^ monocytes participate in the inflammatory response^14,25,26^. During liver inflammation, classical Ly6C^high^ monocytes migrate rapidly to sites of injury, mainly in a CCR2-dependent manner, where they differentiate into functional macrophages and exert either a pro-inflammatory or anti-inflammatory function with the assistance of local environments^25–27^. Interestingly, among F4/80^low^CD11b^+^ monocytes in the liver, the Ly6C^low^ subset of monocytes participates in the resolution of liver injury and tissue repair, whereas the Ly6C^high^ subset of monocytes presents a pro-inflammatory phenotype^11–13^. However, resident F4/80^high^CD11b^+^ macrophage subsets and their functional phenotypes have not been investigated in the liver.

F4/80^high^CD11b^+^ Kupffer cells or resident macrophages play crucial roles in immune tolerance during the normal steady state and also in the inflammatory state^18^. In the non-inflammatory state, Kupffer cells remove detrimental materials such as injured cells, microbes, toxic materials, and immune complexes, to prevent excess immune response and maintain homeostasis^1^. On the contrary, Kupffer cells secrete pro-inflammatory cytokines and chemokines such as TNF-α, IL-1β, and CCL2, due to excessive stimulation by endotoxins or exotoxins^18^. However, whether particular subsets of the Kupffer cell population have pro-inflammatory or anti-inflammatory characteristics is still unclear. Moreover, tissue-resident macrophages might originate from circulating Ly6C^high^ monocytes in the inflammatory state in severe infection, tumor environment, and tissue regeneration^25^. The present results support the suggestion that CX_3_CR1 is an important factor for the differentiation of circulating Ly6C^high^F4/80^low^CX_3_CR1^low^ monocytes into inflammatory CX_3_CR1^high^F4/80^high^Ly6C^low^ macrophages (a new subset), while pre-existing F4/80^high^Ly6C^low^ Kupffer cells are negative for CX_3_CR1 in the steady state^5,28^. After depletion of Kupffer cells by clodronate liposome treatment, considerable numbers of repopulated F4/80^high^CD11b^+^ macrophages were CX_3_CR1 positive (approximately 13%) compared to those (approximately 2.4%) in control CX_3_CR1^+/*GFP*^ mice, whereas they were significantly reduced in CX_3_CR1 deficient mice (approximately 6%). LSECs line the liver sinusoids and provide a platform for the adhesion of diverse immune cells, including Kupffer cells^29^. However, the exact molecular mechanisms by which circulating monocytes differentiate into resident macrophages remain unclear. We observed that LSECs definitely augment the differentiation of circulating blood and splenic monocytes into resident CX_3_CR1^high^F4/80^high^Ly6C^low^ or CX_3_CR1^high^CD14^+^CD16^+^ macrophages, and partially assign pro-inflammatory characteristics to resident macrophages in a CX_3_CR1-CX_3_CL1-dependent manner in both mice and humans. These functional and phenotypical features allow the separation of the two subsets of F4/80^high^CD11b^+^ macrophages in the liver. In terms of CX_3_CL1, there are two different forms of CX_3_CL1. Soluble CX_3_CL1 generated by proteolytic cleavage has a chemoattractive function. Membrane-bound CX_3_CL1 functions as an adhesion molecule^30^. Although we could not examine soluble CX_3_CL1 of HUVECs, siRNA-mediated depletion of CX_3_CL1 in HUVECs decreased the expression of pro-inflammatory genes in adherent human CD14^+^CD16^−^ monocytes. Recombinant CX_3_CL1 treatment reversed the expression of the pro-inflammatory genes. These results suggest that both membrane-bound CX_3_CL1 and soluble CX_3_CL1 can change the function of CX_3_CR1^+^ monocytes. However several studies have reported that CX_3_CR1^high^ macrophages generally have anti-inflammatory characteristics in the intestine but CX_3_CR1^int^Ly6C^high^ cells show pro-inflammatory phenotype in an inflamed colon^31,32^. Therefore, further studies of CX_3_CR1 expressing macrophages are required.

Alcoholic liver disease is one of the main causes of chronic liver disease, in which hepatic macrophages play a critical role during the progression of alcoholic liver disease^33–36^. The number of macrophages and the circulating levels of CCL2 are increased by high endotoxin lipopolysaccharide (LPS) levels in patients with alcoholic liver disease^37–39^. These findings suggest that infiltrated macrophages originating from circulating monocytes could be important in alcoholic liver disease. In support of this idea, Wang *et al*. reported that chronic alcohol consumption can recruit pro-inflammatory Ly6C^high^ or anti-inflammatory Ly6C^low^ cell subsets of F4/80^low^ monocytes^40^. However, the F4/80^high^ subset of macrophages has not yet been investigated. Interestingly, increased serum CX_3_CL1 level correlates with chronic liver diseases, such as liver fibrosis, and CX_3_CR1 regulates the differentiation and survival of infiltrating monocytes in the liver of mice to limit fibrosis^20^. Therefore, the interplay between CX_3_CR1 and CX_3_CL1 might affect the progression of alcoholic liver disease. However, intriguingly, we demonstrated an opposing and detrimental effect of CX_3_CR1 in alcoholic liver disease compared to non-alcoholic liver disease, where CX_3_CR1 accelerated hepatic fat accumulation and liver injury were accompanied by the increased frequency of the pro-inflammatory F4/80^high^CD11b^+^ macrophage subset. These contradictory roles of CX_3_CR1 might be due to the differential activation of macrophage subsets during liver injury. In contrast to the infiltration of large numbers of F4/80^low^CD11b^+^ monocytes in non-alcoholic liver injury, resident F4/80^high^CD11b^+^ macrophages, including Kupffer cells, are the predominant inflammatory cells in alcoholic liver injury and are sensitized by up-regulated endotoxin levels in portal circulation^41^. In a human endotoxemia model, endotoxin treatment increased the frequency of non-classical CD14^+^CD16^+^ monocytes in the blood, due to endotoxin-mediated conversion of circulating classical CD14^high^CD16^−^ monocytes into CD14^+^CD16^+^ monocytes^42^. This human study strongly indicates a possible mechanism of endotoxin-mediated differentiation of circulating classical monocytes into resident-like phenotype of macrophages. However, further studies are required for definitive conclusions.

## Conclusion

CX_3_CR1 plays important roles in the phenotypic conversion and pro-inflammatory functional polarization of hepatic monocytes. CX_3_CR1 could be a specific marker for separating resident macrophage subsets and may be a novel therapeutic target in alcoholic liver disease.

## Materials and Methods

### Animals

Male wild-type (WT) mice with a C57BL/6 or B6/SJL (CD45.1) background and CX_3_CR1 knock-in mice with green fluorescence protein (GFP) (CX_3_CR1^GFP/GFP^) were purchased from the Jackson Laboratory (Bar Harbor, ME). CX_3_CR1^+/GFP^ mice with GFP substitution in only one CX_3_CR1 allele were used as reporters to identify CX_3_CR1-expressing monocytes/macrophages. GFP-expressing CX_3_CR1^GFP/GFP^ mice were considered a s CX_3_CR1 knock-out mice^43^. Male WT mice with a C57BL/6 or B6/SJL (CD45.1) background were used for the generation of chimeric mice. All animals were maintained according to the guidelines of the Care and Use of Laboratory Animals published by the National Institutes of Health. The protocol for animal experiment was approved by Institutional Animal Care and Use Committee of the Korea Advanced Institute for Science and Technology. All mice were euthanized under anesthesia (Ketamine, Yuhan, Korea), then blood, liver, and spleen were harvested.

### Ethanol-induced liver injury

For binge ethanol drinking model, 4 g/kg ethanol was administered to mice by gavage. After 12 h the mice were euthanized. To induce chronic ethanol-induced liver injury, mice were fed a 5% Lieber-DeCarli ethanol liquid diet (Dyets Inc., Bethlehem, PA) for 8 weeks. The liquid diet was replaced daily to prevent evaporation of ethanol. After 8 weeks, the mice were euthanized and liver injury was evaluated.

### Isolation of mouse liver non-parenchymal cells

Liver non-parenchymal cells were isolated as previously described^44,45^. Briefly, the liver was washed with EGTA solution (Sigma Aldrich, St. Louis, MO) and digested with type 1 collagenase (Worthington Biochemical Corporation, Lakewood, NJ) through the portal vein. Hepatic non-parenchymal cells were separated from hepatocytes by centrifugation. HSCs were separated from non-parenchymal cells using Optiprep gradient solution (Sigma-Aldrich, St. Louis, MO). Monocytes and Kupffer cells were isolated using the FACS Aria III device (BD Bioscience, San Jose, CA) and liver sinusoidal endothelial cells (LSECs) were separated using magnetic-activated cell sorting (MACS) with anti-CD146-PE and anti-PE beads (Miltenyi Biotec, San Diego, CA) following manufacturer’s protocol.

### Human samples

Liver tissue of patients with hepatocellular carcinoma and blood of healthy donors were obtained from the archives of the Department of Surgery and the Department of Internal Medicine, Chungnam National University Hospital (Daejeon, South Korea) and the Department of Internal Medicine, Korea University Guro Hospital (Seoul, South Korea), respectively. The study conformed to the ethical guidelines of the Declaration of Helsinki. Authorization for the use of these tissues and blood for research purposes was obtained from the Institutional Review Board of Chungnam National University Hospital (Number: 2016-03-02-003) and Korea University Guro hospital (KUGH16121-001). Informed consent was obtained from all patients who had provided tissue or blood.

### Isolation of human liver mononuclear cells (MNCs) and PBMCs

Human liver MNCs were isolated as previously described^45^ from non-tumor regions of liver tissue from patients with hepatic cellular carcinoma. Liver MNCs were isolated using a GentleMACS™ dissociator (Miltenyi Biotec, Bergisch Gladbach, Germany) according to manufacturer’s instructions. Blood from healthy donors were used for isolation of human PBMCs. The collected blood was centrifuged at 1,600 rpm for 10 min. Plasma was harvested and the buffy coat was suspended in phosphate buffered saline (PBS). The suspended buffy coat was loaded on Ficoll (GE Healthcare, Little Chalfont, UK) and centrifuged for 20 min at 2,000 rpm without brake. PBMCs were harvested from the interphase.

### Clodronate liposome treatment

For depletion of Kupffer cells and resident macrophages, 1 mg clodronate liposomes (Haarlem, Netherlands) were injected intraperitoneally in each mouse. Depletion was confirmed 2 days after the injection. The same concentration of PBS liposomes was injected intraperitoneally as a control.

### Generation of chimeric mice

Chimeric mice were generated as previously described^44,45^. Briefly, the mice were irradiated at dose of a 950 Rad. After irradiation, total BMCs (3 × 10^6^ cells) from donor mice were injected via the tail vein. To deplete the recipient’s Kupffer cells, clodronate liposome treatment was performed 2 days prior to the transplantation of BMCs.

### Co-culture of mouse and human monocytes with mouse LSECs and human umbilical vein endothelial cells (HUVECs)

To differentiate macrophages from monocytes, 2 × 10^5^ monocytes obtained from mouse liver or spleen were co-plated with 5 × 10^5^ LSECs and co-cultured for 24 h. In some experiments, whole liver non-parenchymal cells (5 × 10^5^) were cultured for 24 h. Isolated human CD14^+^ monocytes (5 × 10^5^) from PBMCs were co-cultured with 5 × 10^5^ HUVECs for 48 h. After detachment, co-cultured monocytes were subjected to flow cytometry.

### Flow cytometry

Cells were washed twice with Dulbecco’s PBS containing 0.5% bovine serum albumin and 0.05% sodium azide. After washing, the cells were stained with LIVE/DEAD^TM^ Fixable Aqua Dead Cell Stain Kit (Invitrogen, Eugene, OR) following the manufacturer’s protocol. The cells were stained with fluorescence-conjugated surface antibodies for 30 min at 4 °C, washed, and analyzed using LSR II flow cytometer (BD Bioscience, San Jose, CA). The data were analyzed using FlowJo software (TreeStar, Ashland, OR). Antibodies used for flow cytometric analyses included: APC-Cy7 CD11b (M1/70), APC CD11b (M1/70), PerCP-Cy5.5 Ly6G (1A8), FITC Ly6C (AL21), PerCP-Cy5.5 Ly6C (AL-21), PerCP-Cy5.5 CD45.1 (A20), PE-Cy7 CD3e (145-2C11) PerCP-Cy5.5 CD4 (RM4-5), APC CD8a (53-6.7), and PE NK1.1 (PK136) (all from BD Bioscience); PE-Cy7 F4/80 (BM8), PE F4/80 (BM8) eFluor® 450 CD45 (30-F11) (all from eBioscience, San Diego, CA); PE CX_3_CR1 (polyclonal) and APC CCR2 (#475301) (both from R&D Systems, Madison, WI) for mouse. BV421 CD45 (HI30), PE CD14 (MφP9), and PerCP-Cy5.5 CD16 (3G8) (all from BD Bioscience); FITC CD68 (Y1/82A), APC CX_3_CR1 (FN50), and APC-Cy7 CCR2 (REA264) (all from eBioscience) and APC CD206 (19.2) (Biolegend, San diego, CA) for human.

### Statistical analysis

Data are presented as the mean ± SEM. To compare values obtained from two or more groups, Student’s *t-*test or one-way ANOVA was performed. *P* values < 0.01 or 0.05 were considered statistically significant.

## Electronic supplementary material

Supplementary Information
