# Supplementary material for: CX3CR1 differentiates F4/80low monocytes into pro-inflammatory F4/80high macrophages in the liver
Source: Sci Rep. 2018 Oct 10;8:15076. doi: 10.1038/s41598-018-33440-9 (PMC6180058; doi:10.1038/s41598-018-33440-9)
Supplement: Supplementary file 1 — Supplementary Information [file 41598_2018_33440_MOESM1_ESM.pdf]

CX_3CR1 differentiates F4/80^{low} monocytes into pro-inflammatory F4/80^{high} macrophages in the liver

Young-Sun Lee[1,†], Myung-Ho Kim[2,†], Hyon-Seung Yi[3], So Yeon Kim[2], Hee-Hoon Kim[2], Ji Hoon Kim[1], Jong Eun Yeon[1], Kwan Soo Byun[1], Jin-Seok Byun[4,*], Won-Il Jeong[2,*]

Table of Contents

- Supplementary materials and methods
- Supplementary Fig. 1
- Supplementary Fig. 2
- Supplementary Fig. 3
- Supplementary Fig. 4
- Supplementary Fig. 5
- Supplementary Fig. 6
- Supplementary Table 1
- Supplementary Table 2
- Supplementary references

Supplementary materials and methods

Chronic ethanol diet

The Lieber-DeCarli liquid diet containing 5 % ethanol (Dyets Inc., Bethlehem, PA) was placed on each mouse cage for ethanol diet feeding. The liquid diet was kept in a bottle and replaced daily to prevent evaporation of ethanol. At week 8, mouse liver tissue and blood were collected for the evaluation of liver injury. Sectioned liver tissues were subjected to hematoxylin and eosin (H & E) staining, immunohistochemistry, and Oil Red O staining. Serum levels of alanine aminotransferase (ALT), aspartate aminotransferase (AST), and triglyceride were assessed using kits purchased from IDEXX Laboratories (Westbrook, ME).

Generation of chimeric mouse

Chimeric mice were generated as previously described[1]. Briefly, antibiotic-containing water was fed to recipient mice for 7 days before radiation treatment. Next, the mice were irradiated twice at a dose of 475 Rad at 3-h intervals. Six hours after the first radiation, total BMCs (3×10^6 cells) from donor mice were injected via the tail vein. To deplete the recipient's Kupffer cells, clodronate liposome treatment was performed 2 days before BMC transplantation as previously described[2].

Liver non-parenchymal cell isolation

Livers from mice were homogenized and filtered through a 70-μm nylon cell strainer (BD Bioscience, San Jose, CA). After removing debris, hepatic non-parenchymal cells were collected and suspended in 40% Percoll. The cell suspension was gently overlaid onto 70% Percoll and centrifuged at 4°C for 30 min at 120 x*g*. Liver mononuclear cells (MNCs) were collected from the interface. Then, liver MNCs were resuspended in fresh PBS after RBC lysis. The cell suspension was filtered through a 70-μm cell strainer and centrifuged at 42 x*g* for 5 min at room temperature to remove hepatocytes. The supernatant was transferred to a new tube and centrifuged at 400 *g* for 10 min at 4°C. The pellet was resuspended in 6 ml of 11.5 % Optiprep (Sigma-Aldrich, St. Louis, MO, USA), loaded carefully onto 6 ml of 20 % Optiprep, and centrifuged at 1,800 x*g*

for 17 min at 4°C. The cellular fraction in the interface between 11.5% and 20% Optiprep was gently collected[3].

Stimulation of HUVECs with TNF-α

HUVECs were used in passages 4 to 6 and cultured in EGM-2 SingleQuots® medium (Lonza, Walkersville, MD). HUVECs were seeded with a density of 1 × 10^5 cells/cm^2 in 24 well plates. After 24 h, cells were stimulated with 1 ng/ml TNF-α for another 24 h. Absence of mycoplasma contamination was confirmed using e-Myco VALiD Mycoplasma PCR detection kit (iNtRON Biotechnology, Seongnam, Korea) using input genomic DNAs in HUVECs.

Co-culture of mouse and human monocytes with mouse LSECs and human umbilical vein endothelial cells (HUVECs)

To differentiate macrophages from monocytes, 2 × 10^5 monocytes of liver or spleen were co-plated onto 5 × 10^5 of LSECs. The latter cells had been plated 3 h previously. The cells were co-cultured in Roswell Park Memorial Institute (RPMI)-1640 containing 10% fetal bovine serum (FBS) for 3, 6, 12, or 24 h. Whole cells were detached from the plate at each time point using trypsin-EDTA (Invitrogen, Carlsbad, CA) and the monocytes were separated from LSECs using magnetic-activated cell sorting. Isolated monocytes were analyzed by flow cytometry. In some experiments, 5 × 10^5 whole liver non-parenchymal cells were cultured at each time point. Human monocytes were isolated from peripheral blood mononuclear cells (PBMCs) using MagniSort™ human CD14 positive selection kit (Invitrogen) following the manufacturer's protocol. Human monocytes (5 ×10^5) were co-cultured with 5 × 10^5 HUVECs that had been plated 3 h previously. The co-culture was carried out in EGM-2 SingleQuots® medium for 12, 24, and 48 h. The monocytes were subjected to flow cytometry after detachment using trypsin-EDTA.

Isolation of splenic F4/80^{low}CD11b$^+$ monocytes

The spleen was removed and minced into small pieces, which were homogenized and filtered through a 70-μm cell strainer. The cell suspension was centrifuged at 400 xg, 4°C for 5 min. The supernatant was removed and pellets were treated with 5 ml RBC

lysis buffer (Biolegend, San Diego, CA) for 5 min. The cells were suspended in PBS, centrifuged at 400 xg, 4°C for 10 min, and the splenocytes were collected. F4/80^{low}CD11b^{+} monocytes were isolated using FACS Aria III device (BD Bioscience, San Jose, CA).

Silencing of CX$_3$CL1 in HUVECs and treatment with recombinant CX$_3$CL1

HUVECs were stably transfected with small interfering RNA (siRNA) for CX$_3$CL1 using Lipofectamine® RNAiMAX reagent (Invitrogen) and the EGM-2 BulletKit (Lonza, Walkersville, MD) according to the transfection protocol. For siRNA transfection, we selected the most efficient target sequence provided by Ambion (Austin, TX) using sense 5′- CAAAGAUACCUGUAGCUUUtt -3′ antisense 5′- AAAGCUACAGGUAUCUUUGat -3′. HUVECs were seeded at a density of 2 × 10^5 in a 6-well plate in an EGM-2 BulletKit. CX$_3$CL1 siRNA (25 pmole) or scrambled siRNA were allowed to form duplexes with 9 µl Lipofectamine® RNAiMAX reagent in 300 µl Opti-MEM medium (Gibco, Carlsbad, CA). Cells were transfected and incubated at 37°C with 5% CO_2 for 24 h. CX$_3$CL1-specific siRNA knock-down was assessed by quantitative reverse transcription PCR (qRT-PCR), western blot using antibodies to β-actin (Sigma-Aldrich St. Louis, MO), CX$_3$CL1 (Thermo Fisher Scientific, Waltham, MA) and enzyme-linked immunosorbent assay using Human CX$_3$CL1/Fractalkine DuoSet ELISA DY365 (R&D Systems, Minneapolis, MN) After successful knock down of CX$_3$CL1 in HUVECs using siRNA, human monocytes were co-cultured with transfected or scrambled HUVECs. Some cells were treated with recombinant CX$_3$CL1 (10 ng/ml, R&D Systems). After co-culturing for 12 h, monocytes were separated from HUVECs using Magnisort and subjected to qRT-PCR.

Cytospin and Giemsa staining

Cells (10^5) were diluted in PBS containing 1% bovine serum albumin and pipetted into a sample chamber assembled with glass slides and filter cards. Cells were centrifuged at 400 *g* for 5 min. After checking the number of cells on the slides, the cells were fixed with 10% formalin for 20 min. The fixed cells were dried overnight and then stained with Hema 3 (Fisher Scientific, Hampton, NH)

Tissue staining

Four-micrometer-thick liver tissue sections from paraffin-embedded blocks were stained with hematoxylin and eosin (H & E). Immunohistochemistry was performed with anti-F4/80 antibody (Abcam, Cambridge, UK). Immune complexes were visualized using the ABC kit (Vector Laboratories, Burlingame, CA) and DAB (Invitrogen, Eugene, OR). Immunofluorescence staining was performed with anti-F4/80 antibody (Dako, Glostrup, Denmark) and visualized with Alexa Fluor®594 conjugated anti-mouse IgG secondary antibody (Invitrogen). Finally, Vectashield mounting medium with DAPI (Vector Laboratories, Burlingame, CA, USA) was applied as per the manufacturer's protocol. To visualize lipid droplets in hepatocytes, 7-μm-thick frozen liver sections were stained with Oil Red O solution (Sigma-Aldrich). The sections were visually inspected using a model Olympus BX51 microscope (Olympus, Tokyo, Japan) equipped with a CCD camera and computer-assisted image analysis with DP2-BSW (Olympus) at various magnifications.

cDNA synthesis and qRT- PCR

Sorted or collected cells and liver tissue were treated with TRIzol (Invitrogen) for extraction of total RNA. cDNA was synthesized from RNA with amfiRivert cDNA synthesis master mix (GenDEPOT, Barker, TX) following the manufacturer's protocol. qRT-PCR was performed with SYBR Green real-time PCR master mix (Toyobo, Osaka, Japan) using the CFX96 system (Bio-Rad, Hercules, CA). Supplementary Table 1 and 2 list the PCR primers. The expression of each gene was compared with those of *β-actin* and *GAPDH* and analyzed using ΔΔCt values.

Blood biochemistry

Serum was collected and assayed for ALT, AST and triglyceride (TG) using kits purchased from IDEXX Laboratories (ME, USA).

Gene expression omnibus dataset

To analyze the expression of genes in mouse livers with alcoholic steatohepatitis and alcoholic hepatitis, gene expression data were obtained from the National Center for Biotechnology Information (NCBI) Gene Expression Omnibus (GEO) database

(accession number GSE97234) and previously published high-throughput sequencing data[4]. As previously described[5], Upper Quartile normalization was performed to normalize all samples of read counts per gene/transcript, and Partek Gene Specific Analysis method was used to analyze differential expression. For each comparison, differentially expressed genes (DEGs) were defined as a fold-change >1.0 in either direction and a false discovery rate of 0.05. Among DEGs, heatmaps for *Cx3cr1, Cx3cl1, Il1b,* and *Tnf* were generated using GraphPad Prism (version 7.00; GraphPad Software Inc., La Jolla, CA).

Supplementary Figures

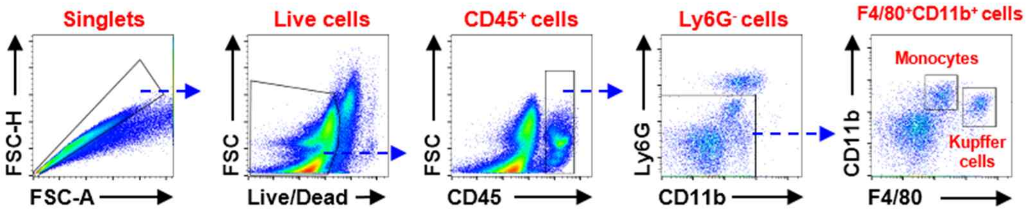

Supplementary Fig. 1. Flow cytometric gating strategy for liver monocytes and Kupffer cells. Liver non-parenchymal cells were isolated and gated for singlets, live cells, $CD45^{+}$cells, and $Ly6G^{-}$ cells before analysis using F4/80 and CD11b antibodies. Monocytes were $F4/80^{low}CD11b^{+}$ and Kupffer cells were $F4/80^{high}CD11b^{+}$.

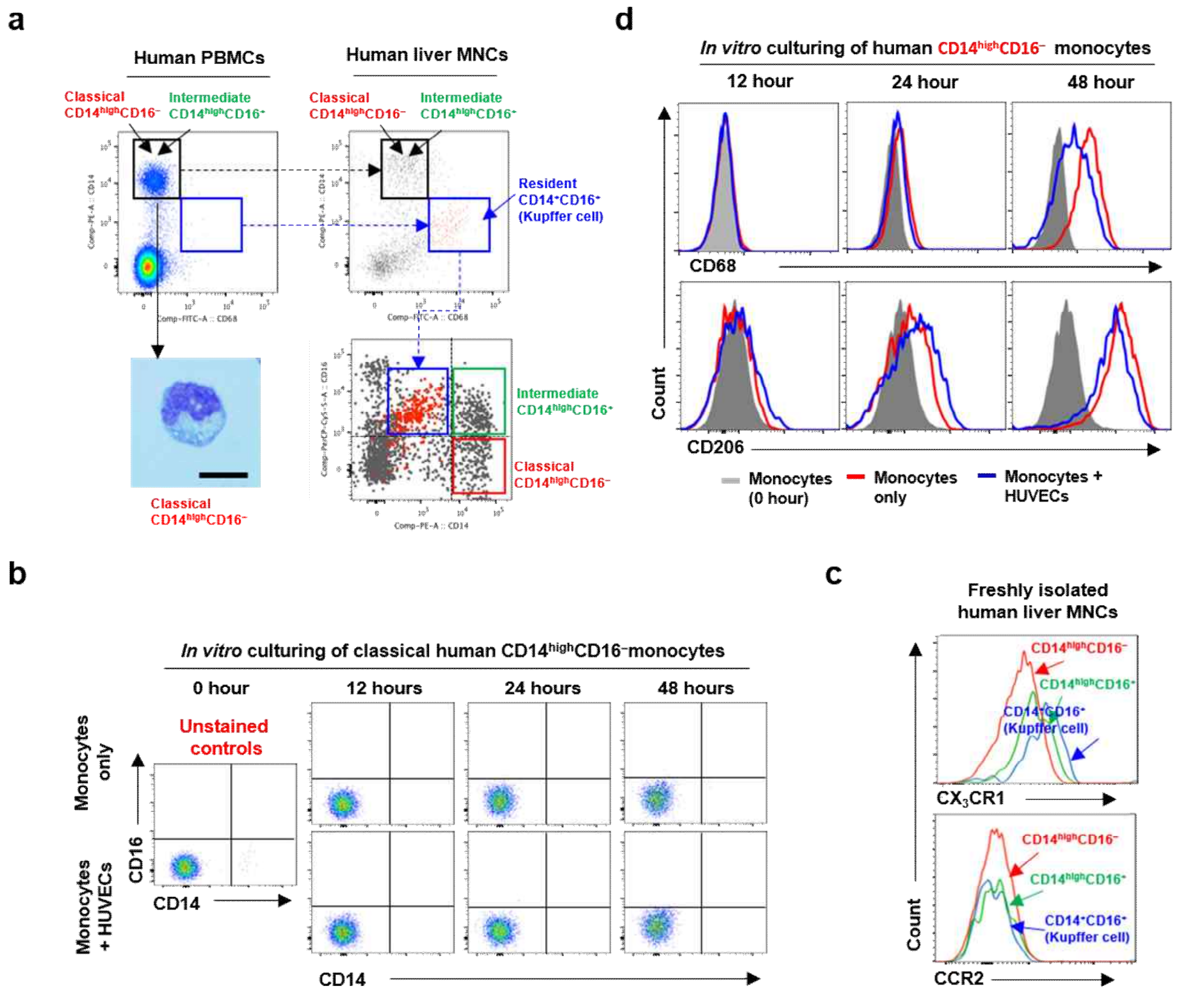

Supplementary Fig. 2. HUVECs differentiate human CD14^{high}CD16$^{–}$ monocytes into CD14^{+}CD16^{+} monocytes along with high expression of CX$_3$CR1. (a) Specific monocyte subsets were compared between human liver MNCs and peripheral blood mononuclear cells (PBMCs). Bar = 10 μm. (b) Human CD14^{high}CD16$^{–}$ monocytes were co-cultured with CX$_3$CL1-expressing HUVECs for 48 h. Flow cytometry analysis in the absence of antibodies was conducted for the unstained control. (c) Expression of CX$_3$CR1 and CCR2 in human liver MNCs was analyzed by flow cytometry. (d) Expression of CD68 and CD206 in co-cultured CD14^{high}CD16$^{–}$ monocytes was analyzed by flow cytometry. The results represent three independent experiments.

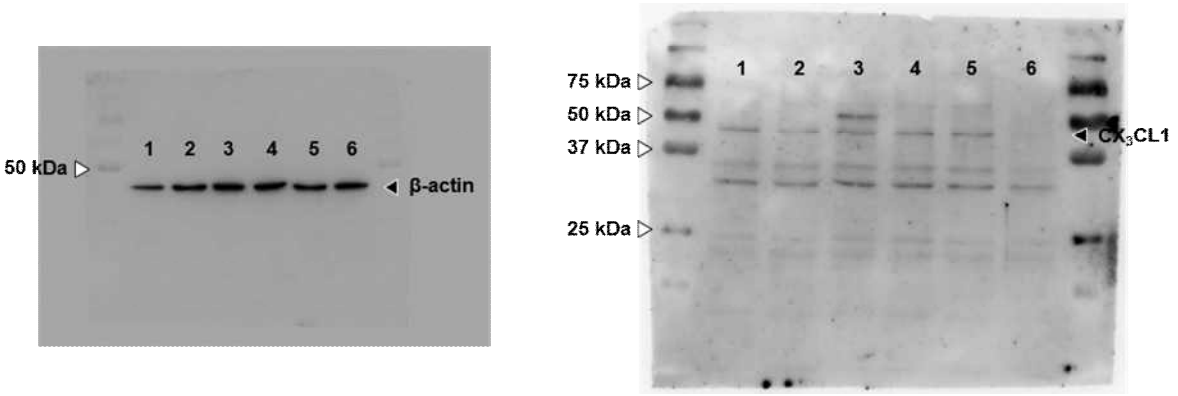

Supplementary Fig. 3. Original western blots for Figure 4a. Lane 4 to 6 were original data of Figure 4a. Lane 4: Control, Lane 5: Scramble, Lane 6: siCX_3CL1. Lane 1 to 3 were not shown in manuscript.

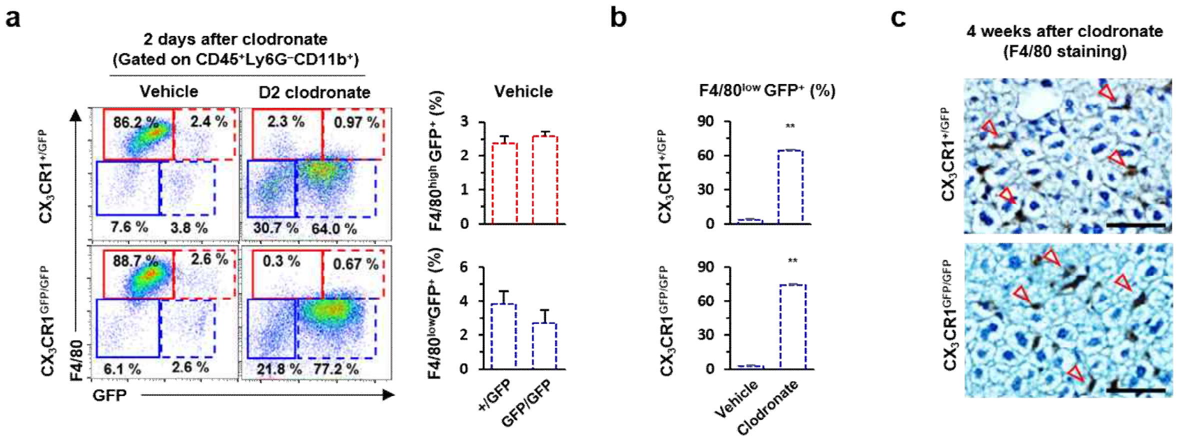

Supplementary Fig. 4. CX_3CR1 expression assigns two subsets and pro-inflammatory phenotype to F4/80^{high} or Ly6C^{low} macrophages in the liver. (a, b) Liver monocytes and macrophages were analyzed by flow cytometry using isolated liver MNCs of $CX_3CR1^{+/GFP}$ and $CX_3CR1^{GFP/GFP}$ mice 2-day after clodronate liposome treatment. Data are expressed as the mean ± SEM. *$p < 0.05$, **$p < 0.01$ compared to the corresponding control. (c) At week 4 after clodronate liposome treatment, liver sections were stained with F4/80 antibody. Arrow heads indicate F4/80$^+$ cells in the liver. Bar = 50 μm. The results represent two independent experiments.

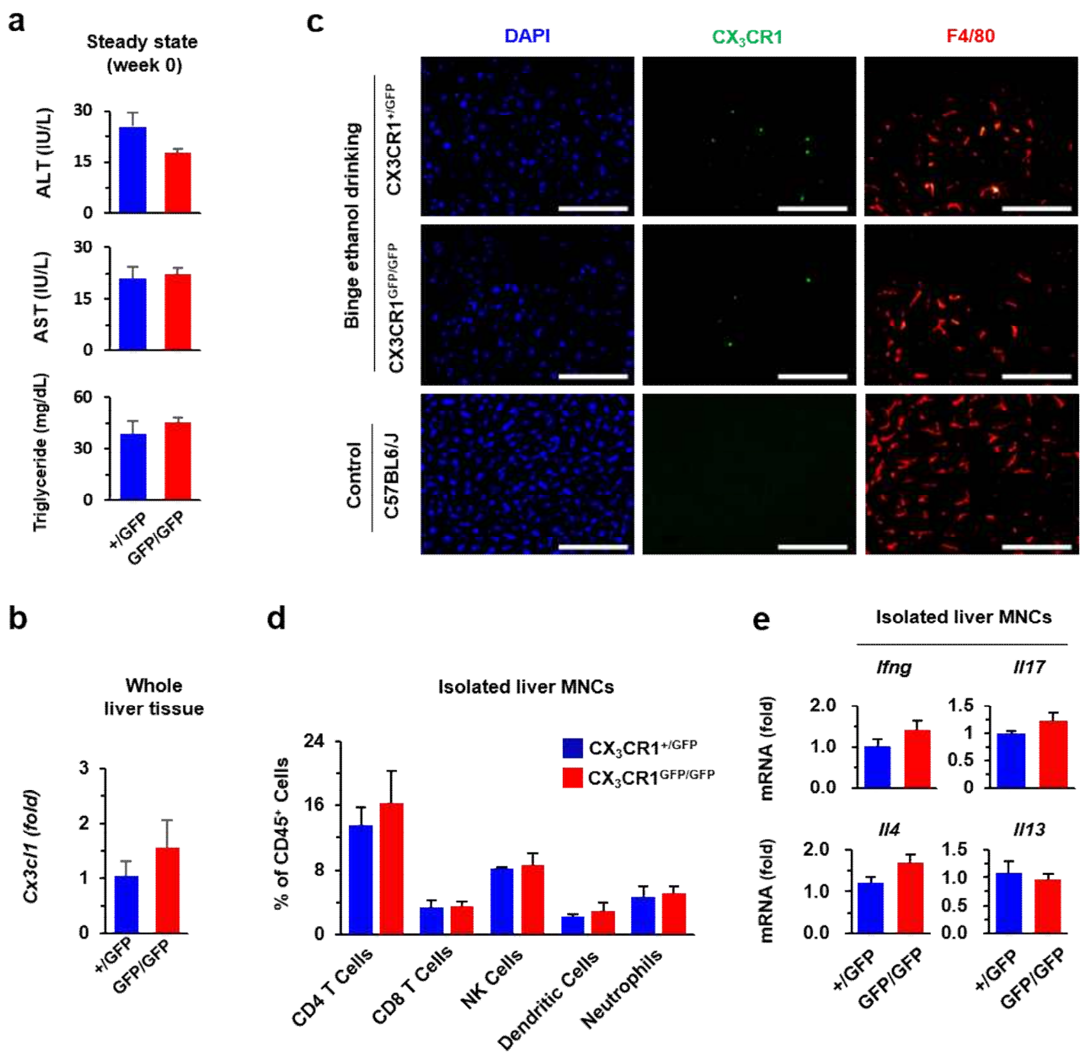

Supplementary Fig. 5 CX3CR1 deficiency attenuates alcoholic liver injury. (a) Serum levels of ALT and AST and triglyceride were measured in steady state $CX_3CR1^{+/GFP}$ and $CX_3CR1^{GFP/GFP}$ mice. (b) Whole liver tissues from binge ethanol-fed mice were subjected to qRT-PCR analysis. (c-e) $CX_3CR1^{+/GFP}$ (n = 4) and $CX_3CR1^{GFP/GFP}$ (n = 4) mice were fed 5% liquid ethanol diet for 8 weeks. (c) Liver sections were stained with DAPI and F4/80 antibody. Bar = 100 µm. (d) Flow cytometric quantification of CD4 T cells, CD8 T cells, NK cells, dendritic cells, and neutrophils. (e) Isolated liver MNCs were subjected to qRT-PCR analysis. Data are expressed as the mean ± SEM. The results represent two independent experiments.

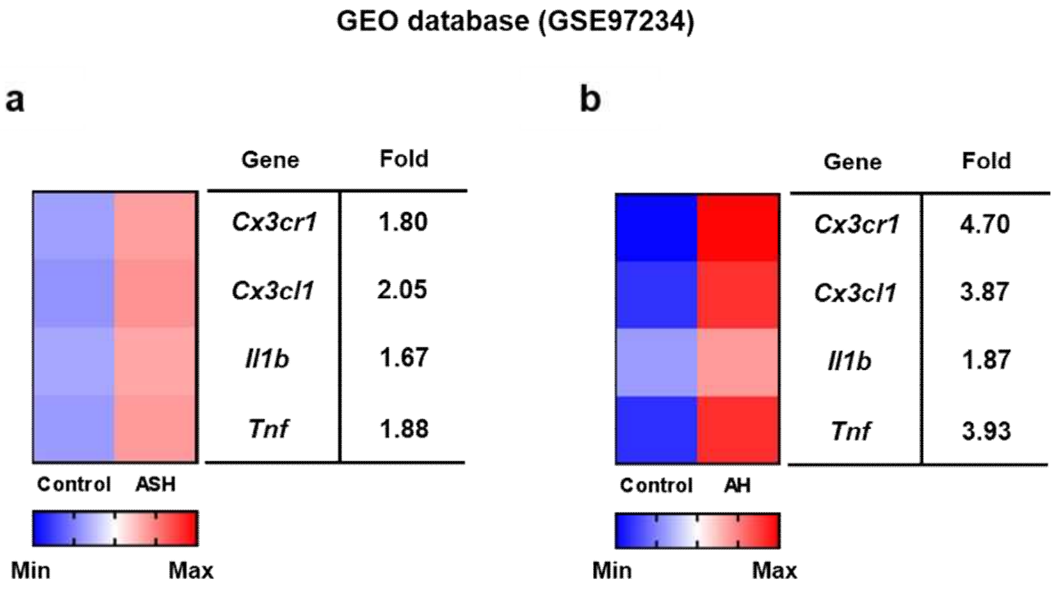

Supplementary Fig. 6. Comparison of gene expression between healthy controls and mice with alcoholic liver disease. Using the GEO database (GSE97234), relative gene expressions of *Cx3cr1, Cx3cl1, Il1b, Tnf,* were compared between healthy control mice and mice with alcoholic steatohepatitis (a) or alcoholic hepatitis (b).

Supplementary Table 1. Primers for real-time PCR (for mice)

Genes	Forward (5’-3’)	Reverse (5’-3’)	PCR product (base pairs)
Arg1	CTCCAAGCCAAAGTCCTTAGAG	AGGAGCTGTCATTAGGGACATC	185
Cx3cr1	CGGCCATCTTAGTGGCGTC	GGATGTTGACTTCCGAGTTGC	126
Cx3cl1	ACGAAATGCGAAATCATGTGC	CTGTGTCGTCTCCAGGACAA	120
Ccl2	TCAGCCAGATGCAGTTAACGC	TCTGGACCCATTCCTTCTTGG	84
Il1b	GCCCATCCTCTGTGACTCAT	AGGCCACAGGTATTTTGTCG	191
Il6	TCCATCCAGTTGCCTTCTTG	TTCCACGATTTCCCAGAGAAC	166
Tgfb1	TTGCTTCAGCTCCACAGAGA	TGGTTGTAGAGGGCAAGGAC	182
Tnf	AAGCCTGTAGCCCACGTCGTA	AAGGTACAACCCATCGGCTGG	140
Actb	AGAGGGAAATCGTGCGTGAC	CAATAGTGATGACCTGGCCGT	148
Ifng	TAGCCAAGACTGTGATTGCGG	AGACATCTCCTCCCATCAGCAG	158
Il17	GCTCCAGAAGGCCCTCAGA	CTTTCCCTCCGCATTGACA	139
Il4	GGTCTCAACCCCCAGCTAGT	GCCGATGATCTCTCTCAAGTGAT	102
Il13	CCTGGCTCTTGCTTGCCTT	GGTCTTGTGTGATGTTGCTCA	116

Supplementary Table 2. Primers for real-time PCR (for humans)

Genes	Forward (5’-3’)	Reverse (5’-3’)	PCR product (base pairs)
CX3CL1	CCTGTAGCTTTGCTCATCCACTATC	TCCAAGATGATTGCGCGTT	69
CX3CR1	ACTTTGAGTACGATGATTTGGCT	GGTAAATGTCGGTGACACTCTT	177
IL1B	ACGATGCACCTGTACGATCA	TGGAGGTGGAGAGCTTTCAG	100
CCL2	GCAGCAAGTGTCCCAAAGAA	GGTGGTCCATGGAATCCTGA	103
TNF	CCTCAGCCTCTTCTCCTTCC	TTAGAGAGAGGTCCCTGGGG	113
ARG1	AATCCTGGCACATCGGGAATC	GTGGAAACTTGCATGGACAAC	76
TGFB1	TACAGCAACAATTCCTGGCG	AAGCCCTCAATTTCCCCTCC	136
IL10	AAGCTGAGAACCAAGACCCA	AAGAAATCGATGACAGCGCC	101
GAPDH	CAGCCTCAAGATCATCAGCA	GTCTTCTGGGTGGCAGTGAT	143

References

1 Yi, H. S. *et al.* Alcohol dehydrogenase III exacerbates liver fibrosis by enhancing stellate cell activation and suppressing natural killer cells in mice. *Hepatology (Baltimore, Md.)* **60**, 1044-1053, doi:10.1002/hep.27137 (2014).

2 Seki, E. *et al.* CCR2 promotes hepatic fibrosis in mice. *Hepatology (Baltimore, Md.)* **50**, 185-197, doi:10.1002/hep.22952 (2009).

3 Kim, S. Y. *et al.* Pro-inflammatory hepatic macrophages generate ROS through NADPH oxidase 2 via endocytosis of monomeric TLR4-MD2 complex. *Nat Commun* **8**, 2247, doi:10.1038/s41467-017-02325-2 (2017).

4 Khanova, E. *et al.* Pyroptosis by caspase11/4-gasdermin-D pathway in alcoholic hepatitis in mice and patients. *Hepatology (Baltimore, Md.)* **67**, 1737-1753, doi:10.1002/hep.29645 (2018).

5 Bullard, J. H., Purdom, E., Hansen, K. D. & Dudoit, S. Evaluation of statistical methods for normalization and differential expression in mRNA-Seq experiments. *BMC Bioinformatics* **11**, 94, doi:10.1186/1471-2105-11-94 (2010).
